# Navigating uncertainty following paediatric disorders of consciousness

**DOI:** 10.1111/dmcn.16280

**Published:** 2025-02-20

**Authors:** Lorna Wales

**Affiliations:** ^1^ Brunel University London Health Sciences Uxbridge UK

## Abstract

**This commentary is on the original article by Chen et al. on pages** 1042–1052 **of this issue.**

Disorders of consciousness (DoC) are an infrequent but impactful consequence of severe acquired brain injury in childhood. DoC, described as an altered state of consciousness, includes coma, vegetative state, and minimally conscious state (MCS). In the UK, it is estimated that each year 350 children and young people fall into the category of probable severe acquired brain injury requiring rehabilitation.^1^ Some of these children will fall into the category of DoC in the short or longer term.

There are three potential outcomes of DoC. Some patients remain in a persistent state for a prolonged period, some experience increased awareness to an MCS level, and some emerge to a fully alert state. However, a recent systematic review concluded that data regarding recovery trajectories in children was inconclusive or contradictory.^2^

One of the critical issues in paediatric brain injury rehabilitation is coping with the uncertainty that arises from the lack of clarity in the trajectory of recovery for those in DoC. In the study by Chen et al., the rate of children who emerged from DoC was less than previously reported.^3^ Only 54% of the cohort regained consciousness at 1 year. The uncertainty of predicting those who will remain in DoC creates a challenge for families and their clinical teams, which can lead to individuals feeling fearful, anxious, and overwhelmed.

Families are navigating a new world, and some families find setting goals and engaging in the rehabilitation programme almost impossible when the prognosis is so uncertain. Some families opt out of shared decision‐making and leave plans to the professionals. Relationships with clinical professionals can become strained, and families can mistrust team members if they are unable to provide accurate predictions. In addition, there are times when the multidisciplinary team feel ill‐equipped to provide information when there is poor data to go on.

Families must make complex decisions when caring for a child with a potentially life‐limiting condition. Frameworks for parallel planning of rehabilitation and palliative care, such as those by Together for Short Lives, can help.^4^ In practical terms, the stakes are high. Major life adjustments like adapting your home, determining and organizing educational supports, sourcing care packages, and considering your work demands cannot be considered lightly. Not only do they take time to navigate, but they are costly to the family and other stakeholders. For example, it would not be uncommon for a major adaptation to a property in the UK to take 18 months to 2 years to complete. In this case, any delays in decision‐making will have an impact on the interim care arrangements for the child, and result in prolonged disruption to family life. However, the possibility of long‐term disability is not certain. It is understandable that a family would be reluctant to embark on such an undertaking if it was not absolutely necessary.

Building trust and compassion with families is crucial, as is respectful, shared decision‐making.^5^ Rehabilitation teams should provide information collaboratively and create accessible narratives that avoid absolutes. There is a fine balance of being pragmatic and initiating processes that are known to be lengthy and bureaucratic, while still maintaining hope.

Chen et al. present an example of using mainly clinical notes to examine the recovery trajectories of children over a number of years.^3^ The data contained many of the elements recommended by Molteni et al.^2^ Such routine data collection within clinical rehabilitation centres worldwide is an achievable goal in a field where large cohort research studies are challenging and costly. International collaboration between clinicians and researchers may yet yield greater benefit.

## FUNDING INFORMATION

None.

## CONFLICT OF INTEREST STATEMENT

The author declares no conflicts of interest.

## Data Availability

Not required.
